# Anti-osteoporotic drugs affect the pathogenesis of gut microbiota and its metabolites: a clinical study

**DOI:** 10.3389/fcimb.2023.1091083

**Published:** 2023-07-04

**Authors:** Rui-kun Zhang, Kun Yan, Hai-feng Chen, Yang Zhang, Gui-jin Li, Xiao-gang Chen, Lin-pu Ge, Feng Cheng, Zhi-neng Chen, Xin-miao Yao

**Affiliations:** ^1^ The Third Clinical Medical College of Zhejiang Chinese Medical University, Zhejiang Chinese Medical University, Hangzhou, Zhejiang, China; ^2^ Department of Orthopedics, The Third Affiliated Hospital of Zhejiang Chinese Medical University, Hangzhou, Zhejiang, China

**Keywords:** gut microbiota, 16S rDNA, untargeted metabolism, osteoporosis, clinical study

## Abstract

**Background:**

Disordered gut microbiota (GM) structure and function may contribute to osteoporosis (OP). This study explores how traditional Chinese medicine (TCM) intervention affects the structure and function of the GM in patients with OP.

**Method:**

In a 3-month clinical study, 43 patients were randomly divided into two groups receiving conventional treatment and combined TCM (Yigu decoction, YGD) treatment. The correlation between the intestinal flora and its metabolites was analyzed using 16S rDNA and untargeted metabolomics and the combination of the two.

**Results:**

After three months of treatment, patients in the treatment group had better bone mineral density (BMD) than those in the control group (*P* < 0.05). Patients in the treatment group had obvious abundance changes in GM microbes, such as Bacteroides, Escherichia-Shigella, Faecalibacterium, Megamonas, Blautia, Klebsiella, Romboutsia, Akkermansia, and Prevotella_9. The functional changes observed in the GM mainly involved changes in metabolic function, genetic information processing and cellular processes. The metabolites for which major changes were observed were capsazepine, Phe-Tyr, dichlorprop, D-pyroglutamic acid and tamsulosin. These metabolites may act through metabolic pathways, the citrate cycle (TCA cycle) and beta alanine metabolism. Combined analysis showed that the main acting metabolites were dichlorprop, capsazepine, D-pyroglutamic acid and tamsulosin.

**Conclusion:**

This study showed that TCM influenced the structure and function of the GM in patients with OP, which may be one mechanism by which TCM promotes the rehabilitation of patients with OP through the GM.

## Introduction

Osteoporosis (OP) has become a nonnegligible disease affecting the quality of life of the elderly (Qaseem et al., 2017). It has an insidious onset and is characterized by low bone mass and destruction of bone structure, which impairs bone strength and leads to an increased risk of fracture (Johnston and Dagar, 2020). Because OP does not receive much attention, many patients suffer secondary fractures that lead to serious complications, resulting in the waste of medical resources (Kanis et al., 2020). The gut microbiota (GM) ecosystem is considered to be a system in the human body (Qin et al., 2010). An increasing number of studies have investigated the microenvironment to explain the relationship between the GM and OP. The maintenance of bone health requires normal bone resorption and good bone formation. It has been reported that the GM is directly or indirectly involved in maintaining normal activity of the gastrointestinal tract and maintaining normal bone health (Espinoza et al., 2016; He et al., 2020). Relevant studies have shown that the GM can promote normal cell metabolism, maintain bone health, and inhibit calcium loss in bone (Schepper et al., 2020; Ling et al., 2021; Zhang et al., 2021b; Guan et al., 2022). A large cohort study of 1776 people in China demonstrated that the GM structure in patients with OP is altered, and its metabolites may be involved in the pathogenesis of OP (Ling et al., 2021).

At present, research efforts on drugs used to treat OP are focused on the field of bone metabolism. There are three main types of drugs that target OP: drugs that promote bone formation, drugs that inhibit bone resorption, and drugs that promote bone mineralization (Srivastava and Deal, 2002; Wang et al., 2009; Coughlan and Dockery, 2014). Although the efficacy of the above drugs is definite, it is still unclear whether they affect the pathogenesis of OP by affecting the composition of the GM or the metabolites of the GM. Most of the recent research has been based on animal experiments that mainly explore the relationship between the GM and OP (Li et al., 2019; Cooney et al., 2020; Schepper et al., 2020; Shen et al., 2021; Hong et al., 2022). Among these studies, research on natural medicine used to treat OP has received increasing attention (Wang et al., 2020; Li et al., 2021; Li et al., 2022b). There are only a few studies on how changes in the GM and its metabolites affect bone metabolism in OP patients after they receive drugs for OP (Lambert et al., 2017). Previous studies have shown that the traditional Chinese medicine (TCM) Yigu decoction (YGD) can treat OP by regulating the expression of proteins in bone tissue (Zhang et al., 2021a), and its efficacy in the treatment of OP has been confirmed in clinical studies (Chen et al., 2021b). To more intuitively study how the GM affects the pathogenesis of OP, we believe that results obtained by investigating the GM of patients with OP would be more convincing. Multi omics can be used to identify and validate molecules involved in the development of diseases influenced by the GM and metabolites. 16S rDNA can be used to identify characteristic nucleic acid sequences and reveal biological species and is considered to be the most suitable method for identifying bacterial phylogeny and taxonomy (Woo et al., 2008). Untargeted metabolomics can be used to investigate the link between identified metabolites and biological processes or biological states when performed using samples and can reveal statistically significant differential metabolites between different populations (Chen et al., 2021a).

Microbiome data can be used to identify the differences in the structure of the GM based on the differences in the abundance of GM microbes, and this analysis can be used to predict or annotate the differences in the functions of the GM. The metabolome is a direct reflection of the interaction between the GM and the host, and the two complement each other. Therefore, the combined analysis of the microbiome and metabolome can enable a better understanding of how the environment of the microbiome affects the metabolic state of the organ environment or host through microbial metabolism and microbial co-metabolism with the host. Therefore, we developed a hypothesis and designed a clinical trial. It was hypothesized that there are beneficial changes in the structure of the GM after the administration of drugs targeting OP. We enrolled 43 patients with OP and randomly divided them into two groups. They were given different treatments, and the GM was evaluated 3 months after treatment. Then, they were analyzed to explore the mechanism by which GM affects OP. It is expected to provide a new direction for the treatment of OP.

## Materials and methods

### Ethics statement, informed consented and clinical trial registration

A total of 43 patients who met the diagnostic criteria for OP were enrolled in this study and randomly divided into two groups between June 2020 and December 2021 at the Department of Orthopedics, The Third Affiliated Hospital of Zhejiang Chinese Medical University. The study was approved by the ethics committee of The Third Affiliated Hospital of Zhejiang Chinese Medical University (approval No. ZSLL-KY-2021-017-01). Moreover, the study has been submitted for registration in the Chinese clinical trial registry using the following registration number: ChiCTR2200056265. All participants provided written informed consent before inclusion in the study.

### Participants and study design

The participants met the diagnostic criteria for a diagnosis of primary OP provided by the guidelines of the National Osteoporosis Foundation (Cosman et al., 2014). We included patients with a vertebral bone mineral density T-score ≤ -2.5 measured by dual-energy X-ray absorptiometry (DXA). Exclusion criteria were as follows: 1) patients with secondary OP; 2) patients with comorbid severe chronic functional disease; 3) patients who had been treated with bisphosphonates, denosumab, and calcitonin within 3 months; and 4) patients with digestive system or liver or kidney function diseases.

### Sample collection and evaluation of clinical parameters

A total of 43 patients were included in the study, and their ages ranged from 71-87 years. Basic information such as the age, height, and weight of all participants was collected before treatment, and there was no significant difference in general characteristics between the two groups (*P >0.05*). Both groups were given 1 tablet of α-Calcitol (0.5 μg/capsule) orally per day for 3 months, and the treatment group was additionally given YGD orally for 3 months. Bone mineral density (BMD) measures were collected before starting treatment and 3 months after treatment, and fecal samples were collected at 3 months after treatment. All patients had fecal samples taken while in the fasted state and at similar time points in the morning.

### Sequencing and bioinformatics analysis

Fecal samples were collected in sterile plastic cups and stored at -80°C before further processing. Total genomic DNA from samples was extracted using the SDS method. DNA concentration and purity were evaluated on 1% agarose gels. According to the concentration, DNA was diluted to 1 ng/µL using sterile water. Genomic DNA extraction and PCR amplification were performed using fecal samples, and PCR products were pooled and purified. Finally, sequencing libraries were generated using the TruSeq^®^ DNA PCR-Free Sample Preparation Kit (Illumina, USA) following the manufacturer’s recommendations, and index codes were added. The library quality was assessed on a Qubit@ 2.0 Fluorometer (Thermo Scientific) and Agilent Bioanalyzer 2100 system. Finally, the library was sequenced on an Illumina NovaSeq platform, and 250 bp paired-end reads were generated. The sequencing data were processed to obtain the effective tags (ET), and the uparse algorithm was used to cluster all the effective tags obtained from all samples, followed by operational taxonomic unit (OTU) clustering and species annotation; finally, alpha diversity and beta diversity were calculated, and functional annotation was performed.

### Analysis of the association between untargeted metabolomic and GM data

Samples stored in a -80°C freezer were thawed on ice. A 400 μL solution (methanol: water = 7:3, V/V) containing an internal standard was added to a 20 mg sample, and the mixture was vortexed for 3 min. Each sample was sonicated in an ice bath for 10 min, vortexed for 1 min, and then placed at -20°C for 30 min. Each sample was then centrifuged at 12000 rpm for 10 min (4°C). The sediment was removed, and the supernatant was centrifuged at 12000 rpm for 3 min (4°C). A 200 μL aliquot of supernatant was obtained for Liquid chromatography–mass spectrometry (LC MS) analysis. All samples were analyzed by the LC MS system following the manufacturer’s instructions. The analytical conditions were as follows: UPLC: column, Waters ACQUITY UPLC HSS T3 C18 (1.8 µm, 2.1 mm*100 mm); column temperature, 40°C; flow rate, 0.4 mL/min; injection volume, 2 μL; solvent system, water (0.1% formic acid): acetonitrile (0.1% formic acid); gradient program, 95:5 V/V at 0 min, 10:90 V/V at 11.0 min, 10:90 V/V at 12.0 min, 95:5 V/V at 12.1 min, 95:5 V/V at 14.0 min.

Unsupervised PCA (principal component analysis) was performed by the statistics function prcomp using R software (www.r-project.org ). The data were unit variance scaled before unsupervised PCA was performed. The HCA (hierarchical cluster analysis) results obtained during the analysis of the samples and metabolites were presented as heatmaps with dendrograms, while Pearson correlation coefficients (PCCs) between the levels of molecules in the samples were calculated by the cor-function in R and presented as only heatmaps. Both HCA and PCC calculation were carried out by the R package Complex Heatmap. Identified metabolites were annotated using the Kyoto Encyclopedia of Genes and Genomes (KEGG) Compound database (http://www.kegg.jp/kegg/compound/ ), and annotated metabolites were then mapped to the KEGG Pathway database (http://www.kegg.jp/kegg/pathway.html ). Significantly enriched pathways were identified using a hypergeometric test p value in the analysis of a specific list of metabolites.

### Statistical analysis

Regarding demographic and clinical parameters, two-tailed t tests were used for paired analyses (before and after intervention) by using the Statistical Package for the Social Sciences (SPSS) version 25.0 (SPSS Inc., Chicago, IL, United States). For the GM data, Wilcoxon’s signed-rank test was used for comparisons of paired samples, and the Mann Whitney U test was used to evaluate independent samples.

## Results

### General characteristics of participants at baseline

A total of 43 individuals were included in the study. They were randomly divided into two groups with 21 in the treatment group and 22 in the control group. The general characteristics of the two groups did not differ significantly and were comparable. After 3 months of treatment, patients in the treatment group showed significantly more improvements in BMD than those in the control group (**Table 1**).

**Table 1:** General data and BMD after 3 months of treatment in both groups.

group	gender	Age (years)	PAS (score)	Pre-BMD	Aft-BMD
	Male/Female				
Control	5/17	79.77 ± 4.253	160.39 ± 6.543	-2.845 ± 0.222	-2.577 ± 0.148^*^
Treatment	4/17	79.29 ± 3.635	161.99 ± 7.214	-2.814 ± 0.176	-2.262 ± 0.132^*#^

PAS, Physical Activity Scale for the Elderly; Pre-BMD, Pretreatment bone mineral density; Aft-BMD, Bone mineral density after treatment. ^*^ Represents a statistically significant within group difference before and after treatment (P < 0.05). ^#^Represents a statistically significant difference between the groups before and after treatment (P < 0.05)

### Species annotation

**Figure 1:**
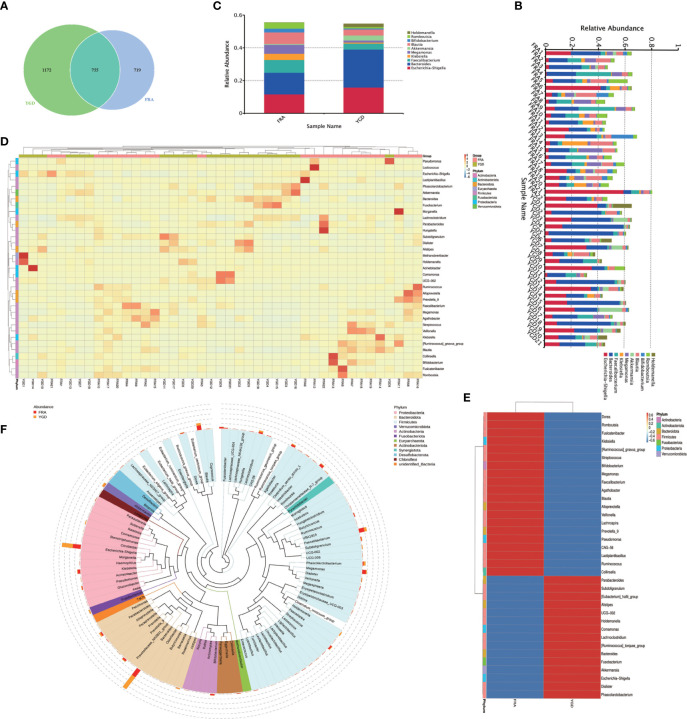
species annotation of the GM in the two groups of OP patients. FRA represents the control group and YGD represents the treatment group. **(A)** Venn diagram based on OTUs. **(B)** The species relative abundance display for each sample at the genus level. **(C)** The top 10 ranked species in abundance at the genus level in both groups. **(D)** Species abundance clustering. **(E)** Species abundance clustering Heatmap. **(F)** Genus level species evolution tree.

Paired-end sequencing of the GM samples obtained from both groups of patients was performed using the Illumina NovaSeq sequencing platform. Tiling and quality control were performed on the resulting data to obtain clean tags, which were then filtered for chimerism to yield effective tags that could be used for subsequent analysis. Clustering of OTUs was performed on the effective tags with 97% concordance (identity), followed by species annotation of the OTUs. A total of 2646 OTUs were identified, of which 755 were common to both groups, 1172 were unique to the treatment group and 719 were unique to the control group (**Figure 1A**). Based on the annotation results, the top 10 species ranked by the maximum abundance at the genus level for each sample were selected, and a column-shaped additive plot of the relative abundance of each species and their ratio in each sample at different taxonomic levels was generated (**Figure 1B**). The top 10 ranked species by maximum abundance at the genus level in each group (Escherichia-Shigella, Bacteroides, Faecalibacterium, Klebsiella, Megamonas, Akkermansia, Blautia, Bifidobacterium, Romboutsia, Holdemanella) are shown in **Figure 1C**. Based on the genus level data on species abundance, the top 35 genera were selected, the abundances of the microbes in each sample were used to cluster the microbes at the species and sample levels, and a heatmap of the species abundance was generated (**Figure 1D**). Of these genera, 15 had significantly more species clustered in the treatment group, and 20 had significantly more species clustered in the control group (**Figure 1E**). To further investigate the phylogenetic relationship of species at the genus level, the representative sequences of the top 100 genera were obtained by multiple sequence alignment (**Figure 1F**). Among them, the top five were Proteobacteria, Bacteroidetes, Firmicutes, Verrucomicrobiota, and Actinobacteria.

### Sample complexity analysis and statistical testing

**Figure 2:**
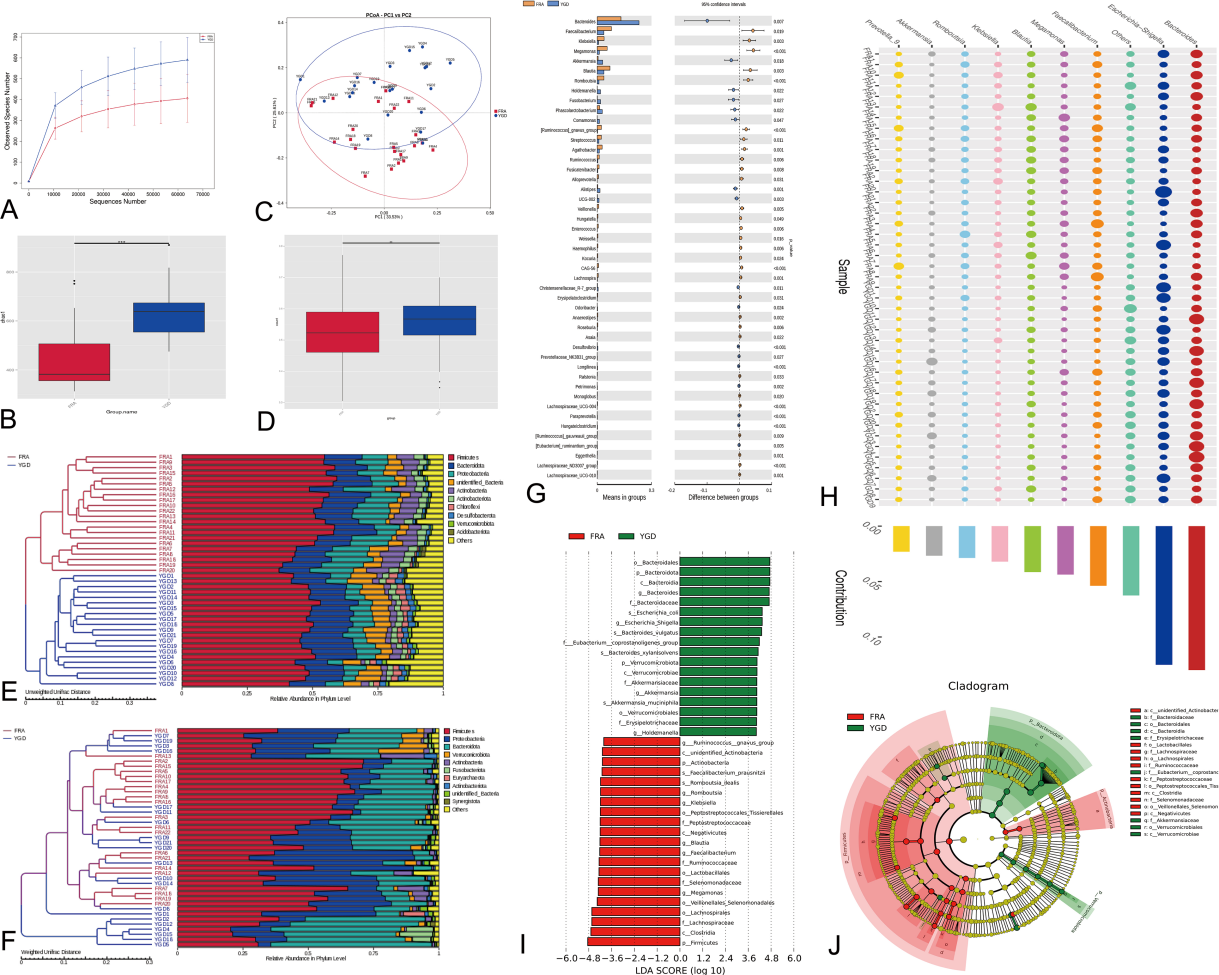
Diversity analysis and statistical tests. **(A)** Species diversity curves. **(B)** Alpha Diversity Index Difference Analysis Between Groups. ***represents *p* < 0.01. **(C)** PCoA analysis based on two groups of species. **(D)** Beta Diversity Index Difference Analysis Between Groups. **represents *p* < 0.05. **(E)** Weighted UniFrac distance matrix UPGMA clustering tree. **(F)** Unweighted Unifrac distance matrix UPGMA clustering tree. **(G)** Differential species analysis between groups (T-test). The figure on the left shows the difference in species abundance between groups. **(H)** Differential species analysis between groups (Simper). **(I)** Differential species analysis between groups (LEfSe) - LDA value distribution histogram. **(J)** Differential species analysis between groups (LEfSe) - Evolutionary branch diagram. Red represents the Biomarker of the different species with obvious changes in the control group, green represents the Biomarker of the different species with obvious changes in the treatment group, and yellow represents the Biomarker of the different species with no obvious difference.

The dilution curve can directly reflect the sufficiency of the amount of sequencing data and indirectly reflect the abundance of species in the sample, and when the curve is flat, the amount of sequencing data is reasonable. The diversity of the microbes within the two patient groups was substantial and homogeneous (**Figure 2A**). The α-diversity index Chao1 was used to determine the ecological diversity within the microbial community. Analyses of the intergroup differences in the alpha diversity index indicated that the two groups of patients had significantly different flora species (**Figure 2B**). We performed principal coordinate analysis (PCoA, principal coordinates analysis) based on the weighted and unweighted UniFrac distances and selected the combinations of principal coordinates with the largest contribution for mapping (**Figure 2C**). The more similar species structure within the two patient groups is indicated in the figure. Box plots of the beta diversity data show significant differences in species between the two patient groups (**Figure 2D**). The multidimensionality confirmed the accuracy of the above results. To investigate the similarity among different samples, clustering trees of the samples can be generated by clustering the samples. The unweighted pair-group method with arithmetic mean (Lü et al., 2019) is a more commonly used method for cluster analysis. UPGMA clustering analysis was performed using the weighted and unweighted UniFrac distance matrices, and the clustering results were displayed by integrating them with the species relative abundances of each sample at the genus level (see **Figures 2E**, **F**). Statistical analysis was used to identify the species with significantly different abundances among the subgroups and evaluate the enrichment of the differential species among the different subgroups (**Figure 2G**). The 193 species with significant differences in abundance at the genus level between the two groups are presented in the figure along with the top 47 differential species. Simper (similarity percentage) is a decomposition of the Bray Curtis dissimilarity index that is used to quantify the contribution of each species to the dissimilarity between two groups (Hamidi et al., 2019). The top 10 ranked species with contributions to the differences between the two groups and their abundances are presented (**Figure 2H**). The top ten species that contributed to the differences belonged to the genera Bacteroides, Escherichia-Shigella, Faecalibacterium, Megamonas, Blautia, Klebsiella, Romboutsia, Akkermansia, and Prevotella_9. Using LEfSe (LDA effect size) to compare the statistical significance and biological correlation of species differences between two groups, we identified biomarkers with significant differences between groups and identified features with different abundances and associated categories. The statistical results of LEfSe were visualized using three approaches: a histogram reflecting the distribution of the LDA value (**Figure 2I**), an evolutionary branch diagram (phylogenetic distribution) and figure illustrating the comparison of the abundance of biomarkers with significant differences between groups in different groups (**Figure 2J**).

### Association analysis, model and functional predictions

**Figure 3:**
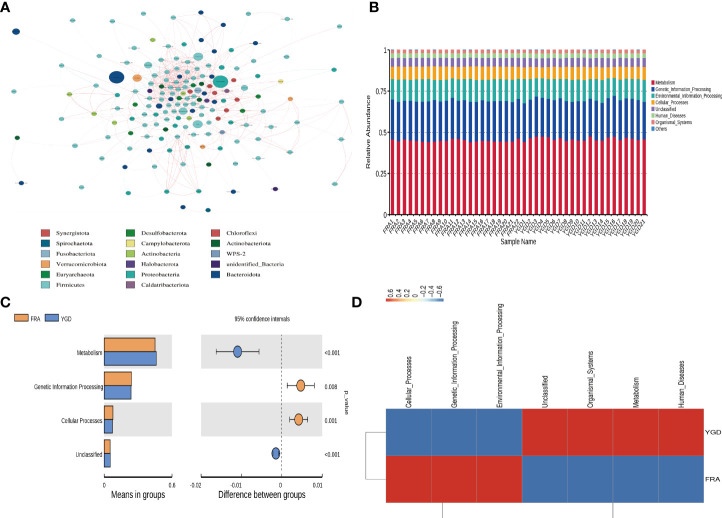
Figure of association analysis, model and functional prediction among species. **(A)** Network analysis. **(B)** Functional annotation relative abundance display. **(C)** T-test for differential function between groups. **(D)** Functional relative abundance cluster analysis.

Network analysis was performed after the data were filtered to yield valid data by performing a correlation index calculation across all samples (**Figure 3A**). Through the species co-occurrence network analysis, we can readily identify the species that dominate the interaction and the species groups that interact closely. These dominant species and species groups may play a unique and important role in maintaining the stability of the microbial community structure and function in the environment. The functional annotation of OTUs obtained from clustering was performed using Tax4Fun. According to the database annotation results, the top 10 functions of each sample or group with the largest abundance at each annotation level were selected, and a columnar stacking chart of the relative abundance of functions was generated to visually display the relative abundance of each microbe at different annotation level functions and their proportions (**Figure 3B**). The top 10 functional information points were analyzed and tested, and the functional information with significant differences was identified (**Figure 3C**). Based on the functional annotation and abundance information obtained from the samples using the database, the top 35 ranked features by abundance and their abundance information in each sample were selected to draw a heatmap and clustered at the level of functional differences (**Figure 3D**). The results showed that there were 4 functional clusters in the treatment group, and the enrichment of 3 functional clusters was significantly decreased.

### Bioinformatic analysis of metabolites

**Figure 4:**
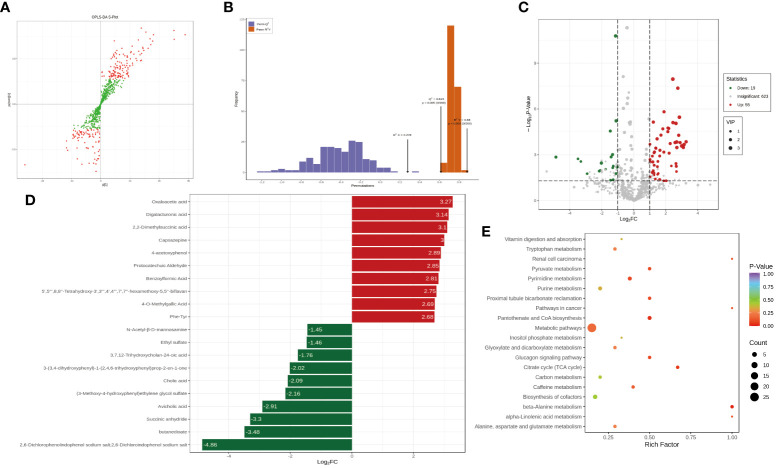
The analysis of the difference of metabolites. **(A)** The orthogonal partial least squares discriminant analysis (OPLS-DA) - S-plot figure. **(B)** OPLS-DA model validation. **(C)** Differential metabolite volcano plot. **(D)** Differential metabolite bar plot. **(E)** Differential metabolite KEGG enrichment analysis.

To further investigate the differences in the sample metabolites between the treatment and control groups, after quantitatively identifying the statistically significant differential metabolites between different groups, we next aimed to investigate the link between the identified metabolites and biological processes or biological states by untargeted metabolomics. Then, the results were analyzed (**Figures 4A, B**) and indicated that the best model interpretation was obtained. Based on the OPLS-DA results, after the different varieties or differential metabolites between tissues were initially identified, the *p* value obtained through univariate analysis was combined to further identify the differential metabolites and draw a volcano plot of the results (**Figure 4C**). Fifty-five of the differential metabolites had higher levels and 19 had lower levels in the treatment group than in the control group, and 623 metabolites had no significant difference in levels. After qualitative and quantitative analysis of the detected metabolites, the fold changes in metabolite levels between groups were compared in addition to the grouping of specific samples (**Figure 4D**). The top 20 differential metabolites are presented in the figure. KEGG pathway enrichment was performed using the differential metabolites (**Figure 4E**).

### Combined analysis of differential metabolites and the GM

**Figure 5:**
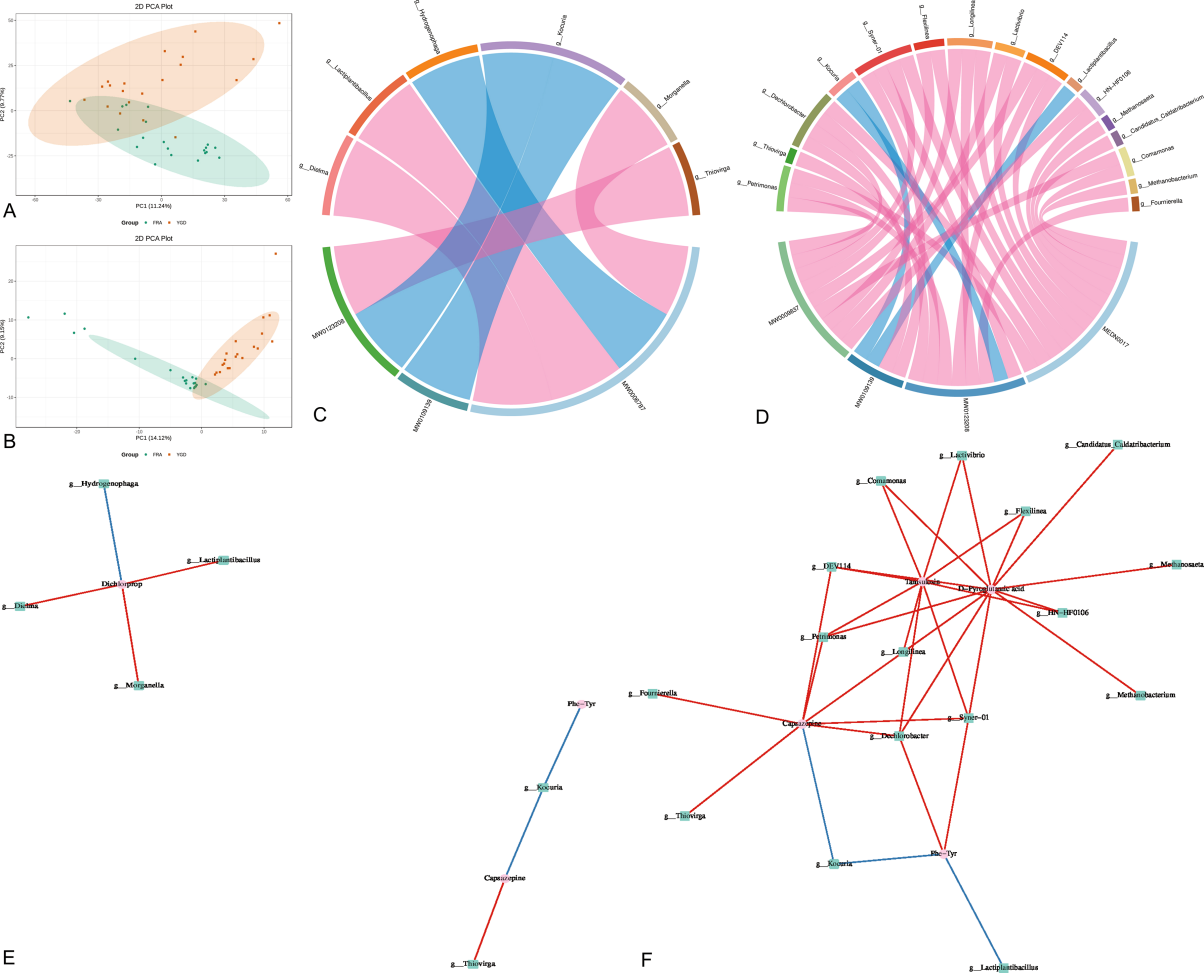
Joint analysis of gut microbiota and differential metabolites. **(A)** Metabolite principal component analysis, score plots of the first 2 PCA principal components. **(B)** Principal component analysis of bacterial groups, score plots of the first 2 PCA principal components. **(C)** Spearman correlation chord plot of differential microbial and differential metabolites. **(D)** Pearson correlation and string plot of differential microbes with differential metabolites. **(E)** Spearman correlation network plots of differential microbes and differential metabolites. **(F)** Pearson correlation network plots of differential microbes and differential metabolites.

To further investigate the connection between the flora and metabolites, we performed a combined analysis of the differences in the patients’ gut microbes and differential metabolites. As shown in **Figure 5A**, the differential metabolites were first subjected to PCA. PCA of the GM microbes was then performed (**Figure 5B**). Differential microbes and differential metabolites were subjected to correlation analysis, and Spearman correlation coefficients were calculated using the levels of microbes and metabolites; significant correlations were defined as those with a correlation |r| > = 0.8 and a *p* - value in the correlation coefficient significance test < 0.05. The results of these analyses of the differential microbes and metabolites are shown in **Supplementary Table 2**. The relevant microorganisms and metabolites identified above were then plotted into a chord plot (**Figure 5C**). This correlation analysis resulted in the identification of significantly correlated microbes and metabolites at the genus level (**Supplementary Table 3**). A chord diagram was drawn (**Figure 5D**). Correlation network plots were generated separately to demonstrate the correlations between microbes and metabolites based on two correlation analysis modalities (**Figures 5E**, **F**).

## Discussion

At present, the number of reports on the effects of the GM on bone metabolism have increased (D’amelio and Sassi, 2018) yearly, and the fact that the GM affects bone metabolism cannot be ignored. It has been reported that the decreased diversity of the GM in OP patients leads to an imbalance in bone homeostasis (Xu et al., 2020). Therefore, probiotics have also gradually begun to be used for the prevention and treatment of OP (Collins et al., 2017; Song et al., 2022). TCM also plays a nonnegligible role in the prevention and treatment of OP (An et al., 2016). According to one report, TCM prevention and treatment of OP can be directly or indirectly involved in bone metabolic pathways affecting bone formation and bone resorption (An et al., 2019; Ren et al., 2020; Xiao et al., 2020); additionally, TCM improves the composition and function of gut bacteria and their metabolic products to achieve the goal of treatment for OP (Wang et al., 2020; Li et al., 2021).

In this study, the investigation of two groups of OP patients with different GM conditions due to intervention and the differences in the metabolites showed that the treatment group improved more than the control group. The BMD in the treatment group improved significantly more than that in the control group; the results also indicate that YGD not only improves BMD in OP patients but also improves the GM, which is indirectly involved in the prevention of OP, confirming the results of our previous study (Li and Gong, 2021; Zhang et al., 2021a). Among the top ten species, Escherichia-Shigella is mainly associated with the inflammatory response and lipid metabolism (Cattaneo et al., 2017; Li et al., 2020). There have also been reports of a correlation between the microbiota and BMD, with side effects suggesting that the microbiota may influence BMD in the lumbar spine and femur, which improves intestinal phosphorus absorption and osteogenic metabolic activity and reduces the excretion of phosphorus (Cheng et al., 2021). The bacilli mainly include single, double, and multi bacilli. Among them, Bacteroides, Faecalibacterium, and Bifidobacterium have all been reported to be involved in the occurrence and development of OP. Previous studies have shown that the abundance of Bacteroides species in normal people is higher than that in OP patients (Wang et al., 2017; Tang et al., 2021), and in our study, the abundance of Bacteroides species in patients in the treatment group was higher than that in the control group, indicating that YGD can improve the abundance of this flora. Faecalibacterium and Bifidobacterium have similarly been reported to have some effects against OP (Xu et al., 2020; Kwon et al., 2021; Lee et al., 2021). In addition, several other flora species have been reported to be potentially involved in the process of OP development (He et al., 2020; Tu et al., 2020; Kwon et al., 2021; Qin et al., 2021). Holdemanella was reported to be associated not only with OP but also with metabolic diseases such as diabetes, which seems to indicate that there may be some connection between OP and diabetes (He et al., 2020; Cheng et al., 2021; Romaní-Pérez et al., 2021). It is worth mentioning that Blautia species were more enriched in the control group (*P* = 0.003) than in the treatment group. The presence of this microbe is considered a predisposing factor for OP; other study results indicated that YGD inhibited this microbe, and the therapeutic effects of YGD were verified (Liu et al., 2020). The GM has functional effects; therefore, we evaluated the functional changes in the GM. The metabolic function of the GM is an important factor affecting OP (Khosla and Hofbauer, 2017). Genetic information processing has also been proven to participate in the disease process of OP as a function of the GM (Xu et al., 2020; Lee et al., 2022). Similarly, the function of GM cellular processes affecting bone metabolism has been explained (D’Amelio and Sassi, 2018, Saxena et al., 2021). These results indicate that the above microbes with distinct changes may be involved in these functions and thus affect the pathogenesis of GM.

Metabolomics is performed by quantifying metabolites from the GM and can be used to identify the relative relationship of metabolites with physio pathological changes. Reports on capsazepine suggest that it may direct bone marrow mesenchymal stem cells toward pro-osteogenic differentiation to combat OP, and the main mechanism may involve transient receptor potential cation channel subfamily V member 1 (TRPV1) intervention (Pan et al., 2013; Xiao et al., 2019). There is no related research on how Phe-Tyr is involved in OP, but it has been reported in a recent study that Phe-Tyr affects the onset of diabetes as a substance affecting glucose metabolism (Jahja et al., 2014; Strasser et al., 2015), which also confirms the finding in our previous study that there may be a relationship between senile OP and diabetes (Zhang et al., 2021a). Phe-Tyr is associated with inflammation of the bone and joint (Muhammed et al., 2020). By performing KEGG pathway enrichment on the differential metabolites, we found that there were several predominant enriched signaling pathways as follows: metabolic pathway, TCA cycle, and beta-alanine metabolism. The TCA cycle is a component of the central metabolic pathways used in all aerobic organisms, including gut microbes (Krebs and Johnson, 1980; Granchi et al., 2019). Dickens first pointed out the close connection between citrate and bone in 1941(Dickens, 1941). An increasing number of reports have confirmed that citrate can contribute to the mineralization of bone cells and provide sufficient energy for the osteogenic differentiation of BMSCs (Franklin et al., 2014; Costello et al., 2015). Basic research has also confirmed the involvement of this metabolite and its metabolic pathway in OP (Si et al., 2020). Similarly, beta alanine metabolism has been reported to be involved in the bone remodeling process (Yu et al., 2019; Li et al., 2022a). This finding sheds light on the fact that starting with metabolites and identifying their targets and pathways that affect OP may be a research direction.

To maintain a stable niche, the interaction between microbial metabolites and host signals influences a variety of metabolic pathways in the host, strongly influences the host metabolic phenotype, promotes the evolutionary adaptation of the host, and promotes a mutually beneficial relationship between the host and microbes. The combined study of the microbiome and metabolome can help us better understand how environmental microbes and their associated metabolism and cometabolism with the host influence the environment within the host or the host metabolic state. Through combined analysis, we found that the metabolites dichlorprop, capsazepine, D-pyroglutamic acid and tamsulosin had the strongest and most positive correlations with microorganisms. However, at present, there are few reports on how the metabolites D-pyroglutamic acid and tamsulosin affect bone metabolism, and further studies may need to confirm their mechanism in OP.

This study explored the role of TCM in improving the GM through different intervention modalities. Through research, we found that some gut microbes, such as Escherichia Shigella, Bacteroides, Faecalibacterium, Bifidobacterium, Blautia and Holdemanella, were changed significantly in OP patients under TCM intervention. The functions of the GM that change significantly are mainly reflected in three processes, including metabolic function, genetic information processing and cellular processes. The main metabolites that have been linked to the development of OP include capsazepine and Phe – Tyr, and they may mainly act through metabolic pathways, the TCA cycle, and beta alanine metabolism. This finding validates previous relevant studies and demonstrates that there may be a certain advantage of TCM interventions targeting the GM. In this study, we also identified differential metabolites with possible connections to OP, including dichlorprop, capsazepine, D-pyroglutamic acid and tamsulosin.

## Conclusion

The composition and function of the GM influence the recovery of patients with OP. In this study, we demonstrated the changes in the GM and microbial metabolites in patients with OP under TCM intervention and their effects on bone metabolism. The effects of TCM intervention on GM were confirmed.

## Data availability statement

The datasets presented in this study can be found in online repositories. The names of the repository/repositories and accession number(s) can be found below: Bioproject and associated SRA metadata, PRJNA988844.

## Ethics statement

The study was approved by the ethics committee of The Third Affiliated Hospital of Zhejiang Chinese Medical University, approved No. of ethic committee: ZSLL-KY-2021-017-01. Meanwhile, the study has been submitted for registration in the Chinese clinical trial registry, registration number: ChiCTR2200056265. All participants provided written informed consent before inclusion in the study. The patients/participants provided their written informed consent to participate in this study. Written informed consent was obtained from the individual(s) for the publication of any potentially identifiable images or data included in this article.

## Author contributions

R-KZ, X-MY and Z-NC conceived and designed the study. R-KZ, KY performed most of the research and wrote the paper, and H-FC participated in the full process of writing the paper. YZ and G-JL performed part of the study and L-PG, X-GC and FC analyzed the data. All authors contributed to the article and approved the submitted version.
