# Supplementary material for: Anti-osteoporotic drugs affect the pathogenesis of gut microbiota and its metabolites: a clinical study
Source: Front Cell Infect Microbiol. 2023 Jul 4;13:1091083. doi: 10.3389/fcimb.2023.1091083 (PMC10354646; doi:10.3389/fcimb.2023.1091083)
Supplement: Supplementary file 1 [file Table_1.docx]

Supplementary Material

# Supplementary Figures and Tables

Table 2 Spearman correlation coefficients between differential microbes and differential metabolites at genus level

| **Compounds** | **Taxonomy** |
| --- | --- |
| Phe-Tyr | k__Bacteria;p__Actinobacteria;c__unidentified_Actinobacteria;o__Micrococcales;f__Micrococcaceae;g__Kocuria |
| Capsazepine | k__Bacteria;p__Actinobacteria;c__unidentified_Actinobacteria;o__Micrococcales;f__Micrococcaceae;g__Kocuria |
| Capsazepine | k__Bacteria;p__Proteobacteria;c__Gammaproteobacteria;o__Halothiobacillales;f__Halothiobacillaceae;g__Thiovirga |
| Dichlorprop | k__Bacteria;p__Firmicutes;c__Bacilli;o__Lactobacillales;f__Lactobacillaceae;g__Lactiplantibacillus |
| Dichlorprop | k__Bacteria;p__Proteobacteria;c__Gammaproteobacteria;o__Enterobacterales;f__Morganellaceae;g__Morganella |
| Dichlorprop | k__Bacteria;p__Firmicutes;c__Bacilli;o__Erysipelotrichales;f__Erysipelotrichaceae;g__Dielma |
| Dichlorprop | k__Bacteria;p__Proteobacteria;c__Gammaproteobacteria;o__Burkholderiales;f__Comamonadaceae;g__Hydrogenophaga |

Table 3 Pearson's correlation coefficients between differential microbes and differential metabolites at genus level

| **Compounds** | **Taxonomy** |
| --- | --- |
| D-Pyroglutamic acid | k__Bacteria;p__Proteobacteria;c__Gammaproteobacteria;o__Burkholderiales;f__Comamonadaceae;g__Comamonas |
| D-Pyroglutamic acid | k__Bacteria;p__Chloroflexi;c__Anaerolineae;o__Anaerolineales;f__Anaerolineaceae;g__Longilinea |
| D-Pyroglutamic acid | k__Bacteria;p__Bacteroidota;c__Bacteroidia;o__Bacteroidales;f__Dysgonomonadaceae;g__Petrimonas |
| D-Pyroglutamic acid | k__Bacteria;p__Synergistota;c__Synergistia;o__Synergistales;f__Synergistaceae;g__Syner-01 |
| D-Pyroglutamic acid | k__Archaea;p__Euryarchaeota;c__Methanobacteria;o__Methanobacteriales;f__Methanobacteriaceae;g__Methanobacterium |
| D-Pyroglutamic acid | k__Bacteria;p__Synergistota;c__Synergistia;o__Synergistales;f__Synergistaceae;g__Lactivibrio |
| D-Pyroglutamic acid | k__Bacteria;p__Chloroflexi;c__Anaerolineae;o__Anaerolineales;f__Anaerolineaceae;g__Flexilinea |
| D-Pyroglutamic acid | k__Archaea;p__Halobacterota;c__Methanosarcinia;o__Methanosarciniales;f__Methanosaetaceae;g__Methanosaeta |
| D-Pyroglutamic acid | k__Bacteria;p__Verrucomicrobiota;c__Verrucomicrobiae;o__Pedosphaerales;f__Pedosphaeraceae;g__DEV114 |
| D-Pyroglutamic acid | k__Bacteria;p__Proteobacteria;c__Gammaproteobacteria;o__Burkholderiales;f__Rhodocyclaceae;g__Dechlorobacter |
| D-Pyroglutamic acid | k__Bacteria;p__Firmicutes;c__Clostridia;o__Oscillospirales;f__Hungateiclostridiaceae;g__HN-HF0106 |
| D-Pyroglutamic acid | k__Bacteria;p__Caldatribacteriota;c__Caldatribacteriia;o__Caldatribacteriales;f__Caldatribacteriaceae;g__Candidatus_Caldatribacterium |
| Phe-Tyr | k__Bacteria;p__Firmicutes;c__Bacilli;o__Lactobacillales;f__Lactobacillaceae;g__Lactiplantibacillus |
| Phe-Tyr | k__Bacteria;p__Actinobacteria;c__unidentified_Actinobacteria;o__Micrococcales;f__Micrococcaceae;g__Kocuria |
| Phe-Tyr | k__Bacteria;p__Synergistota;c__Synergistia;o__Synergistales;f__Synergistaceae;g__Syner-01 |
| Phe-Tyr | k__Bacteria;p__Proteobacteria;c__Gammaproteobacteria;o__Burkholderiales;f__Rhodocyclaceae;g__Dechlorobacter |
| Capsazepine | k__Bacteria;p__Actinobacteria;c__unidentified_Actinobacteria;o__Micrococcales;f__Micrococcaceae;g__Kocuria |
| Capsazepine | k__Bacteria;p__Chloroflexi;c__Anaerolineae;o__Anaerolineales;f__Anaerolineaceae;g__Longilinea |
| Capsazepine | k__Bacteria;p__Bacteroidota;c__Bacteroidia;o__Bacteroidales;f__Dysgonomonadaceae;g__Petrimonas |
| Capsazepine | k__Bacteria;p__Synergistota;c__Synergistia;o__Synergistales;f__Synergistaceae;g__Syner-01 |
| Capsazepine | k__Bacteria;p__Verrucomicrobiota;c__Verrucomicrobiae;o__Pedosphaerales;f__Pedosphaeraceae;g__DEV114 |
| Capsazepine | k__Bacteria;p__Proteobacteria;c__Gammaproteobacteria;o__Halothiobacillales;f__Halothiobacillaceae;g__Thiovirga |
| Capsazepine | k__Bacteria;p__Firmicutes;c__Clostridia;o__Oscillospirales;f__Ruminococcaceae;g__Fournierella |
| Capsazepine | k__Bacteria;p__Proteobacteria;c__Gammaproteobacteria;o__Burkholderiales;f__Rhodocyclaceae;g__Dechlorobacter |
| Tamsulosin | k__Bacteria;p__Proteobacteria;c__Gammaproteobacteria;o__Burkholderiales;f__Comamonadaceae;g__Comamonas |
| Tamsulosin | k__Bacteria;p__Chloroflexi;c__Anaerolineae;o__Anaerolineales;f__Anaerolineaceae;g__Longilinea |
| Tamsulosin | k__Bacteria;p__Bacteroidota;c__Bacteroidia;o__Bacteroidales;f__Dysgonomonadaceae;g__Petrimonas |
| Tamsulosin | k__Bacteria;p__Synergistota;c__Synergistia;o__Synergistales;f__Synergistaceae;g__Syner-01 |
| Tamsulosin | k__Bacteria;p__Synergistota;c__Synergistia;o__Synergistales;f__Synergistaceae;g__Lactivibrio |
| Tamsulosin | k__Bacteria;p__Chloroflexi;c__Anaerolineae;o__Anaerolineales;f__Anaerolineaceae;g__Flexilinea |
| Tamsulosin | k__Bacteria;p__Verrucomicrobiota;c__Verrucomicrobiae;o__Pedosphaerales;f__Pedosphaeraceae;g__DEV114 |
| Tamsulosin | k__Bacteria;p__Proteobacteria;c__Gammaproteobacteria;o__Burkholderiales;f__Rhodocyclaceae;g__Dechlorobacter |
| Tamsulosin | k__Bacteria;p__Firmicutes;c__Clostridia;o__Oscillospirales;f__Hungateiclostridiaceae;g__HN-HF0106 |
